# Current Trends and Future Prospects of Radiomics and Machine Learning (ML) Models in Spinal Tumors—A Narrative Review

**DOI:** 10.3390/jimaging12030138

**Published:** 2026-03-19

**Authors:** Vivek Sanker, Suhrud Panchawgh, Anmol Kaur, Vinay Suresh, Dhanya Mahesh, Eeman Ahmad, Srinath Hariharan, Dhiraj Pangal, Maria Jose Cavgnaro, Mirabela Rusu, John Ratliff, Atman Desai

**Affiliations:** 1 Department of Neurosurgery, Stanford University, Palo Alto, CA 94304, USA; pangal@stanford.edu (D.P.); mjcava@stanford.edu (M.J.C.); jratliff@stanford.edu (J.R.); atman@stanford.edu (A.D.); 2 Department of Neurology, Mayo Clinic, Scottsdale, AZ 85259, USA; 3 Department of Neurosurgery, Lady Hardinge Medical College and Associated Hospitals, New Delhi 110001, India; 4 Department of Neurosurgery, King George Medical University, Lucknow 226003, India; 5 Department of Neurosurgery, Dartmouth Geisel School of Medicine, Hanover, NH 03755, USA; dhanya.mahesh.med@dartmouth.edu; 6 Department of Neurosurgery, Fatima Memorial Hospital College of Medicine and Dentistry, Lahore 54000, Pakistan; 7 Department of Radiology, Stanford University, Palo Alto, CA 94304, USA; hsrinath@stanford.edu (S.H.); mirabela.rusu@stanford.edu (M.R.)

**Keywords:** artificial intelligence, radiomics, deep learning, machine learning, spinal cord tumors

## Abstract

The intersection between radiomics, the computational analysis of imaging data, and machine learning (ML) may lead to new developments in the diagnosis, prognosis, and management of diseases. For spinal tumors specifically, applications of these fields appear promising. In this educational narrative review, we provide a summary of the current advancements in radiomics and artificial intelligence (AI), as well as applications of both fields in the diagnosis and management of spinal tumors. We also provide a suggested workflow of radiomics and machine learning analysis of spinal tumors for researchers, including a list and description of commonly used radiomic features. Future directions in the field of radiomics and machine learning applications to spinal tumors may involve validating already proposed algorithms with larger datasets, ensuring that all computational applications to patient care maintain high ethical standards, and continuing work in developing novel and highly accurate computational techniques to enhance patient outcomes.

## 1. Introduction

Spinal tumors are classified as intradural intramedullary, intradural extramedullary (IDEM), and extradural, based on their anatomical location [1]. Intramedullary lesions, including ependymomas, astrocytomas, and hemangioblastomas, are rare, accounting for 2–5% of all primary tumors of the central nervous system (CNS) [2]. IDEM tumors, including schwannomas and meningiomas, are more common, constituting between 30% of all spine tumors, whereas extradural tumors, mostly metastatic, are the most common, constituting about 60% of all spine tumors [3]. Extradural tumors are generally metastatic and often originate from the vertebral bodies [1]. Metastases to the spine may cause serious complications such as pathological fractures and spinal cord compression, potentially resulting in permanent neurological dysfunction [4,5].

Standard imaging methods, particularly magnetic resonance imaging (MRI), are used for spinal tumor diagnostics, though they are not entirely sensitive in distinguishing spinal tumors from other spinal pathologies. For example, MRI of intramedullary spinal cord lesions and inflammatory demyelinating diseases of the spine, such as multiple sclerosis and neuromyelitis optica (NMO) spectrum disorder, may have indistinguishable features [6]. Spinal multiple myeloma and metastases to the spine also look similar on multiple different imaging sequences; thus, distinguishing and adequately diagnosing spinal lesions can be a difficult [7] and time-consuming process [5,7].

Radiomics is the computational identification and analysis of features from radiological imaging [8]. Deep learning (DL), another type of machine learning (ML), is another type of feature identification and analysis [7]. The application of radiomics and DL to image analysis may improve both diagnostics and personalized treatment for patients with spinal tumors [7,8]. Catching and treating these tumors early can stop tumor spread to other areas of the body and improve a patient’s prognosis [9].

Herein is a narrative and comprehensive review of the current advancements of radiomics and machine learning, as well as applications to the diagnosis and management of spinal tumors. We also provide a suggested workflow in radiomics and machine learning applications for spinal cord tumors. Finally, we will discuss future directions in the field. This review is primarily organized by the anatomic region of the spinal tumor.

## 2. Materials and Methods

As this is a narrative review, we employed a broad search strategy to identify and synthesize studies of interest, including original studies, clinical studies, case reports, and series with no predefined timeframe. To find such studies, we performed a literature search across various databases, namely PubMed, EMBASE, and Scopus. This search utilized precise search terms, including “radiomics”, “machine learning”, “artificial intelligence”, “deep learning”, “radiotherapy”, “chemotherapy”, and “spinal cord tumors”. In addition to electronic searches, a manual search was performed to identify references from recently published series and reports. Abstracts and unpublished studies were excluded to maintain the robustness of the review. A comprehensive summary of the methodology is provided in Table 1 for quick reference, delineating the specifics of the search strategy and inclusion criteria (see Table 1). As this is an educational narrative review, we summarized applicable example articles for this review. While we aimed to be comprehensive, we did not employ systematic review strategies, as this is a narrative review. As such, we did not conduct a formal bias assessment, which may influence our interpretations of the mentioned studies.

## 3. Artificial Intelligence, Machine Learning, and Radiomics: Introduction and Theory

### 3.1. Machine Learning Model Types

Machine learning (ML) represents the aspect of artificial intelligence (AI) through which specified datasets are used to create algorithms that predict or make inferences about new data [10,11]. In spinal tumor research, ML can extract the features of medical imaging that standard radiology evaluation cannot, in order to detect clinically significant patterns in spine imaging studies [11]. ML models can be categorized into four major categories: supervised (labeled datasets), unsupervised (unlabeled datasets), semi-supervised, and reinforcement learning [10]. In supervised learning, the model either performs regression, to predict numerical values, or performs classification, to categorize a particular data point [10,11]. In spinal tumor research, supervised learning may be a method of using radiologist-evaluated imaging labels for model training. For example, Li et al. used a regression-type model to predict loss of blood during surgery [12]. The model was trained on a variety of patient factors with the labels as milliliters of blood loss [12].

Unsupervised learning involves the creation of algorithms to detect inherent patterns without prelabeled outputs [13]. Unlike supervised techniques, statistical methods like clustering and dimension reduction methods are employed to identify patterns in the data [10]. Some of the common clustering algorithms are k-means, hierarchical clustering, and DBSCAN [10]. Applications of unsupervised learning in spinal tumor research may be clustering groups of patients or imaging data to better understand tumor sub-classes, for example.

Semi-supervised learning capitalizes on both labeled and unlabeled sets [10]. Notable semi-supervised methods include self-training, which improves the performance of the model by labeling unlabeled data based on labeled data [14]. Semi-supervised learning may be applicable to researchers planning to use both radiologist-annotated and non-annotated imaging datasets, for example.

Reinforcement learning (RL) uses dynamic feedback from the environment to improve decision-making processes [10,15]. RL builds decision-making policies through a series of iterative interactions, with rewards for actions being beneficial and penalties for suboptimal actions, refining the performance over time [15].

### 3.2. Types of Algorithms

In addition to selecting the type of ML model, the proper selection of specific algorithms can maximize the performance of the model. Artificial neural networks (ANN) are based on biological neural networks, connecting multiple units such that the output of one unit serves as the input of another, thereby transforming multiple inputs into desired outputs [16]. Of note, ANNs work through forward transmission of information, creating a “feed-forward neural network” [16]. Convolutional neural networks (CNNs), another type of DL network, are commonly used as they do not require user-defined features, as they are able to autonomously select features [17]. For example, Arends et al. used CNNs for patients with metastatic spinal tumors, noting the algorithm’s speed and ability to select features from the unprocessed CT images [18].

Other algorithms utilize support vector machines (SVMs), supervised learning techniques used for classification, regression, and identifying outliers [19]. The primary goal of a SVM is to make a decision distinction between two classes, so the model can predict labels from one or more feature vectors [19]. This technique enables the classification of individual observations into distinct classes based on high-dimensional data [19]. The process involves optimizing the algorithm’s parameters using training data and then evaluating its generalization performance using test data [19]. SVM not only requires the training patterns to lie on the correct side of the decision boundary but also necessitates a safety margin for better generalization capability [19]. SVMs may be a potential option for researchers looking to predict either one of two labels using spine tumor patient data.

Decision trees, another algorithm, can be used mainly for classification as well as for regression [20]. Decision trees begin with a root node where the datasets are split into two at the threshold value of the most potent predictor of an outcome [20]. The datasets are subsequently split into various classes at each following node, depending on a single feature [20]. This binary partition of the data at each node is referred to as recursive partitioning [20]. Random forest (RF) algorithms are an extension of decision trees [20]. In an RF, a subsample of features is used to create each decision tree, and results from all the individual trees are combined to produce an accurate and unbiased overall prediction [20]. Massaad et al. used the random forest method to study the frailty of surgical spine tumor patients [21]. The researchers found that random forest performed better than logistic regression [21].

### 3.3. Radiomics

Separate from machine learning, radiomics is a cutting-edge discipline that focuses on extracting and analyzing quantitative features from medical imaging [22]. These features, known as imaging biomarkers, provide detailed information about the characteristics of tissues, tumors, and other pathologies through subtle details about the shape, texture, intensity, and patterns within the images [22]. By providing a deeper understanding of the disease, radiomics can enhance diagnostic accuracy [22]. Furthermore, radiomics can improve treatment planning by identifying the most effective therapeutic approaches and predicting potential complications [23].

### 3.4. Segmentation (Utilizing Radiomics and Machine Learning)

AI and ML applications to the segmentation of spinal tumor imaging have made substantial advancements [24]. Semi-automated segmentation tools powered by deep learning algorithms have been shown to accurately delineate tumor boundaries, an essential step [24]. These AI-driven processes significantly reduce the time and effort required for radiologists, thereby increasing workflow efficiency and accuracy [24]. These types of applications may help streamline spine tumor image processing.

## 4. Workflow Proposed for Radiomics and ML Applications to Spinal Cord Tumors

**Figure 1:**
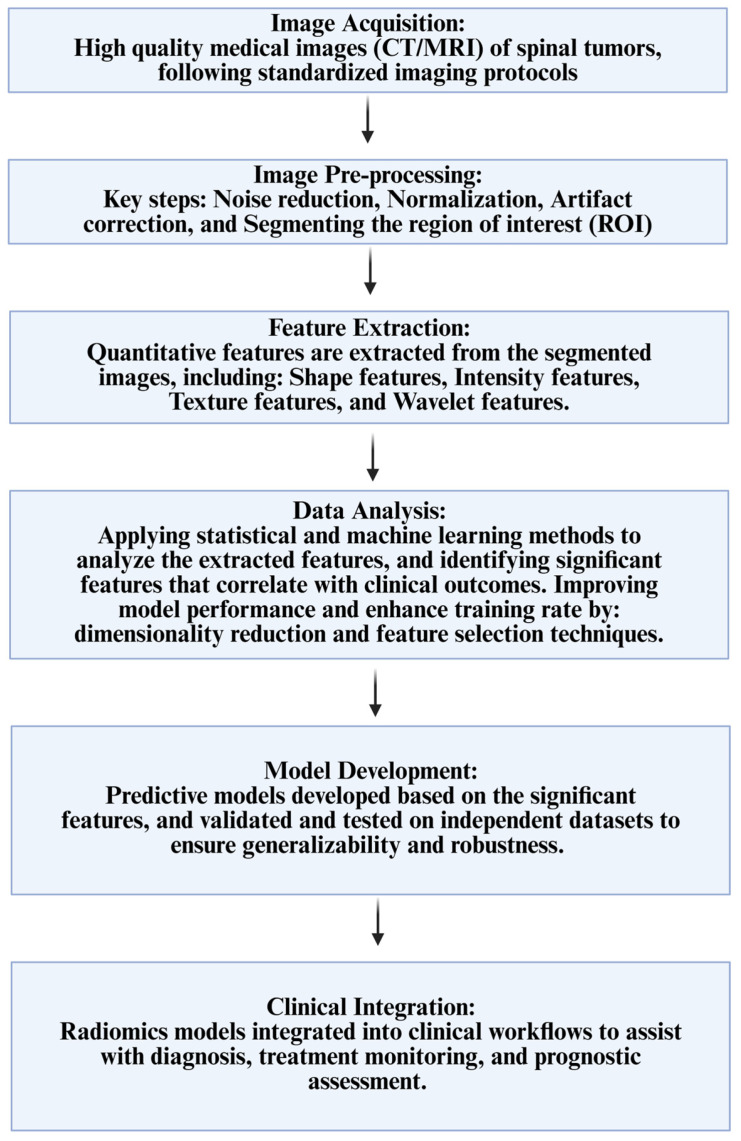
Summary of workflow of radiomics [25,26,27,28].

Based on the studies chosen for this narrative review, we summarize and propose a methodology for AI and ML applications to spinal tumors with the incorporation of radiomics data. Figure 1 summarizes a radiomics-specific workflow to identify, extract, and analyze imaging features through predictive modeling [25,26,27,28].

### 4.1. Proposed Features for Radiomics Analysis

Potential radiomics features for analysis are described below, all summarized from Zwanenburg et al. 2020 [29].

#### 4.1.1. Shape Features

Shape features are quantitative descriptors that provide crucial information about the geometric properties of a region of interest (ROI) within medical images. The primary shape features include:

- Volume: Represents the total size of the tumor in three-dimensional space, calculated by summing the volumes of all voxels within the ROI. This would provide clinicians with an understanding of the size of a tumor.
- Surface Area: Measures the total area of the outer surface of the tumor, providing insights into its boundary complexity and surgical planning.
- Compactness: A dimensionless measure indicating how closely packed the tumor cells are, defined as the ratio of the tumor volume to the volume of a sphere with the same surface area.
- Sphericity: A dimensionless feature describing the roundness of the tumor. This measure, alongside compactness, may provide more information about tumor growth.

#### 4.1.2. Texture Features

Texture features capture the spatial distribution of pixel intensities within an ROI, providing detailed information about tissue heterogeneity. Key texture features include:

- GLCM (Gray-Level Co-Occurrence Matrix): This includes metrics like contrast, correlation, energy, and homogeneity, potentially providing more insight into tumor types or stages.
- GLRLM (Gray-Level Run Length Matrix): Measures the length of consecutive pixels with the same gray-level value, reflecting texture roughness and smoothness, potentially providing some clues into distinguishing benign versus malignant tumors.
- NGTDM (Neighborhood Gray-Tone Difference Matrix): Assesses the difference between a pixel and its neighbors, capturing texture variations by evaluating parameters like coarseness, contrast, and busyness, providing more information about tumor aggressiveness.

#### 4.1.3. Intensity Features

Intensity features are statistical measures of the pixel values within the ROI. They describe the distribution of intensity values, offering insights into the tissue composition. Key intensity features include:

- Mean Intensity: The average intensity value of the pixels within the ROI, providing more information about the tissue of interest.
- Median Intensity: The middle value of the intensity distribution, providing a robust measure less affected by outliers, also providing information about tissue.
- Standard Deviation: Reflects the variation in pixel intensity, indicating the heterogeneity within the ROI. Heterogeneity may provide information about tumor malignancy.
- Skewness: Measures the asymmetry of the intensity distribution, indicating whether the pixel values are more concentrated on one side of the mean.

#### 4.1.4. Wavelet Features

Wavelet features are derived from wavelet transformations, which decompose the image into components at multiple scales, capturing both fine details and overall structures. Key wavelet features include:

- High and Low-Frequency Components: Represent the detailed and approximate information within the image, respectively, providing more information about tumor characteristics.
- Wavelet Decomposition Coefficients: Provide multi-scale information about the image, essential for identifying patterns and structures at different levels of resolution.

## 5. Applications of Radiomics, Artificial Intelligence, and Machine Learning Models in Spine Tumors

In this section, we detail general applications of radiomics, AI, and ML models to spinal tumors. We then provide a discussion of these applications to intradural and extradural spinal tumors, as well as metastases to the spine.

### 5.1. General Operative Risk Assessment and Cost-Effective Treatment Planning

AI/ML models have been studied in pre-operative risk stratification and postoperative function prediction modeling for patients with spinal tumors [30,31]. For example, Liu et al. (2015) used active shape models and optical flow models to pre-operatively plan dose distributions for spine stereotactic body radiation therapy (SBRT) [31]. While this particular study was limited by the sample size (30 patients), by assisting with pre-operative planning, these models may be able to reduce spinal cord injury and overall improve patient outcomes [31]. Other researchers have also created deep learning models and machine learning-based decision-making tools for pre-operative planning for spine radiation therapy [32,33,34]. Some studies have utilized convolutional neural networks (CNNs), but there are noted limitations [18]. For example, Arends et al. (2022) demonstrated that their CNN-based approach could have better time management in manual delineation processes [18]. In terms of postoperative predictors, in a study by Huang et al. (2023), a nomogram was able to estimate the early death risk (postoperative) for older patients who are planned for spinal tumor surgery [30].

Other studies that offer methods to potentially increase the cost-effectiveness of spinal tumor treatment include a study by Hong et al. (2020) on automated SBRT planning for paraspinal tumors using “constrained hierarchical optimization”, which produced SBRT plans in a time-efficient manner [35]. Stieler et al. (2009) explored a “neuro-fuzzy technique” using AI to complete optimization of a radiation treatment plan, potentially improving cost efficiency [36].

Intraoperative decision-making has also benefited from AI advancements. Reinecke et al. (2022) introduced a CNN to predict the existence of a tumor using “stimulated Raman scattering microscopy images” during surgery, potentially reducing the likelihood of incomplete tumor removal or damage to healthy tissue [37]. As previously mentioned, Li et al. (2023) developed a model to predict “intraoperative blood loss” for patients who had undergone surgical resection for metastases to the spine, which offers a robust tool for surgical planning, outperforming traditional ordinary least squares regression models [12].

**Figure 2:**
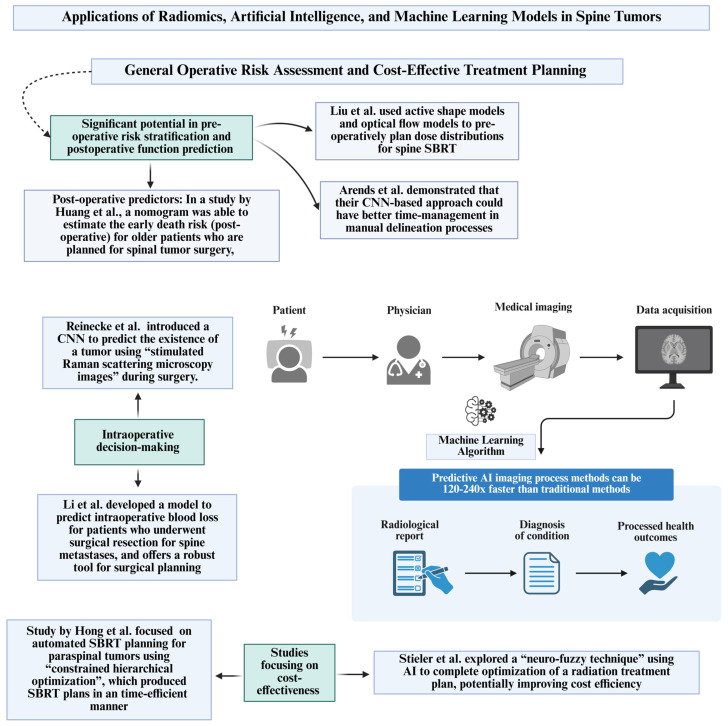
Role of artificial intelligence and radiomics in general operative risk assessment and cost-effective treatment planning with references to published studies Li et al. [12], Arends et al. [18], Liu et al. [31], Hong et al. [35], Stieleret al. [36] and Reinecke et al. [37].

As described above, combining artificial intelligence to analyze pre-operative risk and maximize cost-efficiency may improve the way clinicians treat spinal tumors (Figure 2). Limitations for this field may be inherent bias in using retrospective data to train models [30] and the potential lack of integration of training data from multiple centers [35].

### 5.2. General Spinal Tumor Predictive Modeling for Treatment, Outcomes, and Prognosis

Prognosis and outcomes for patients with spinal tumors are an area of active research. We offer a general summary of research within prognostics and outcomes below, while specifically delineating by spinal tumor type in Section 5.3. For instance, Schoenfeld et al. (2019) analyzed the outcomes of surgical treatment for lumbar metastases, using Bayesian regression to account for confounding variables, and found that surgery is associated with a decreased likelihood of mortality and an increased likelihood of ambulatory capacity [38]. However, the study also noted that surgery may be related to a higher chance of “complications and readmissions”, underscoring the need for careful patient selection [38].

Other recent studies have emphasized the importance of accurate prognostic models to guide treatment decisions and predict patient outcomes. DiSilvestro et al. (2020) demonstrated the utility of a “Naïve Bayes classifier” in assessing 30-day mortality after spine tumor removal [39]. Their model outperformed traditional risk calculators, emphasizing the potential of machine learning algorithms in enhancing predictive accuracy and informing surgical planning [39]. Other studies have used predictive modeling to select good patient candidates for surgery and select treatments for spine tumor patients [40,41]. Models have also been built to use patient characteristics such as frailty [21,42,43] and older age [30,44] to predict outcomes.

Many studies have utilized radiomics specifically to potentially guide treatment and outcomes. For example, Gui et al. (2021) utilized radiomics to accurately identify patients with spinal metastases who may have vertebral compression fractures after undergoing stereotactic body radiation therapy (SBRT) [45]. Additionally, Sanli et al. (2022) explored the use of radiomics in patients with spinal bone metastases to predict survival after 6 months [46], though radiomics itself did not enhance their model [46].

In short, machine learning and radiomics show immense potential in predicting outcomes, treatment and prognosis.

### 5.3. Spinal Tumor Specific Modeling and Radiomics Applications

**Figure 3:**
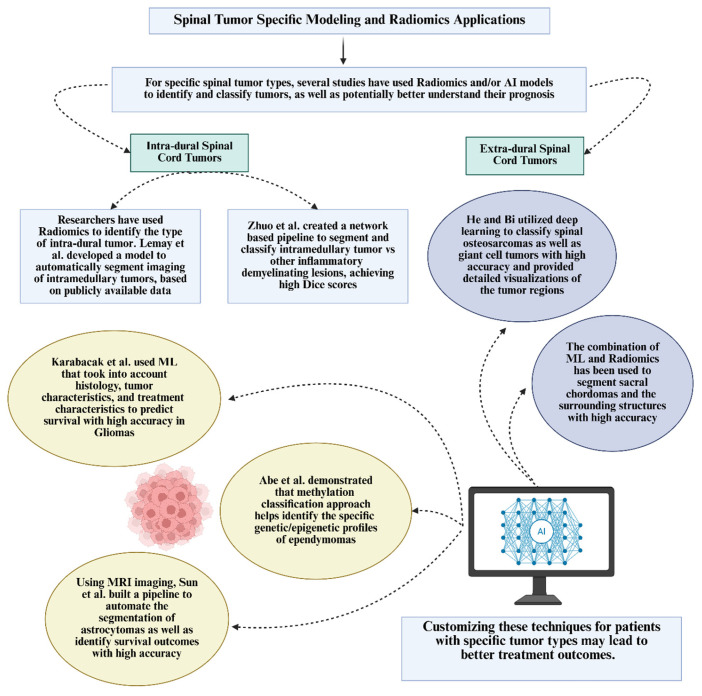
Spinal tumor-specific modeling and radiomic applications with references to published studies Lemay et al. [2], Zhuo et al. [6], Karabacak et al. [47], Sun et al. [48] and Abe al. [49].

For specific spinal tumors, many studies have used radiomics and/or artificial intelligence to identify and classify tumors, as well as potentially better understand prognosis. Bringing these computational techniques into clinical practice could potentially improve healthcare efficiency and patient outcomes. Customizing these techniques for patients with specific tumor types may also lead to better outcomes. This is summarized in Figure 3.

#### 5.3.1. Intradural Spinal Cord Tumors

Researchers have used radiomics to identify types of intradural tumors [2,6]. Lemay et al. (2021) developed a model to automatically segment imaging of intramedullary tumors, which is publicly available [2]. Zhuo et al. (2022) created a network-based pipeline to segment and classify intramedullary tumor vs. another inflammatory demyelinating lesion, achieving high Dice scores [6].

Specific applications within types of intradural tumors are detailed below. Karabacak et al. (2024) used machine learning that took into account histology, tumor characteristics, and treatment characteristics to predict survival with high accuracy [47]. This tool is available publicly as well [47]. Using MRI, Sun et al. (2022) built a pipeline to automate the segmentation of astrocytomas as well as identify survival outcomes with high accuracy [48]. The model’s integration of different types of imaging sequences enhances its predictive power, offering a potential tool for better patient care [48]. Abe et al. (2022) demonstrated that methylation classification could provide a molecular diagnosis that complements traditional histopathological methods [49]. This approach helps identify the specific genetic/epigenetic profiles of ependymomas, offering a more nuanced understanding of tumor behavior and aiding in more accurate diagnosis and treatment planning [49].

#### 5.3.2. Extradural Spinal Tumors

He and Bi (2024) utilized deep learning to classify spinal osteosarcomas as well as giant cell tumors [50]. This model achieved high accuracy and provided detailed visualizations of tumor regions, potentially helping orthopedic surgeons maximize patient care [50]. The combination of machine learning and radiomics has been used to identify skull base chordomas versus chondrosarcomas [51], segment chordomas of the sacral region and the surrounding structures [52] with high accuracy. Machine learning has also been implemented in drug development research for chordoma specifically [53].

#### 5.3.3. Spinal Metastases

Radiomics applications in spinal metastases include the utilization of many different types of imaging, including 3D CT [54], MRI [55], and PET with CT [5]. Radiomics-based modeling has been used to identify spinal metastases versus other pathologies as well [56,57]. Other applications of machine learning and/or radiomics include the prediction of patient outcomes such as mortality [58,59] and intraoperative loss of blood [60].

In terms of outcome prediction, genetic data has also been incorporated into these models. For example, Jiang et al. (2023) used deep learning to identify the EGFR mutation in spine metastases from NSCLC [61]. Other studies have incorporated radiomics to predict EGFR mutations in spine metastases from pulmonary adenocarcinoma [62,63].

## 6. Future Directions

Synthesizing this research field, it is clear that the main challenges will be the implementation of these clinical predictive models and radiomics workflows into clinical practice. To do so, most urgently needed is better interpretability [24], allowing clinicians without a computational background to understand how models make clinical decisions [24]. Other necessary changes include improvements to and expansion of training sets, general guidance for clinicians to make this part of the standard workflow, and ensuring that these models can keep patient data safe and secure [64].

Ethics are important to consider with further integration of radiomics and AI models into the clinical workflow. As noted above, health privacy is important to uphold, as well as support from the appropriate authority groups [64]. Finally, other considerations necessitate that these AI and radiomics models are continuously checked to ensure accuracy and clarity [64].

Finally, these models can be improved with larger datasets [64]. The availability of such datasets may improve the prevalence of external validation in this field. Finally, many of these models require replication studies to ensure that results are accurate. Standardized pipelines, such as the one proposed in Section 4, that are implemented by radiomics and ML researchers at large would improve both validation and replication studies.

Future directions for the field of radiomics and artificial intelligence applications to spinal tumors are numerous and explored below:

- Diagnostics: Utilizing radiomics in medical imaging can provide a more robust number of quantitative features that may help in tumor detection and diagnostics [65]. For example, implementing radiomics in standard radiologist clinical workflows can help understand what quantitative features correlate to particular tumor diagnoses and prognoses.
- Personalized Treatment: Incorporation of radiomics with other clinical characteristics, such as genomic information, can lead to more specific and patient-specific treatment plans, potentially improving treatment outcomes [66]. Creating public repositories of genetic, imaging, and clinical information can lead to more robust machine learning algorithms. The diversity of data used to train these algorithms may lead to more accurate predictions and reveal insights into tumor biology.
- Real-Time Monitoring and Treatment: Incorporation of radiomics in real-time, with the power of vast computational analysis, may allow physicians to adjust patient management in real-time. In this way, patient treatment plans can be updated based on the most recent available clinical results, potentially improving patient outcomes.
- Automation and Accessibility: Automation incorporating radiomics has been shown to be useful within the diagnostics of brain tumors [67]. Further research in this field could improve healthcare accessibility and efficiency [67]. This may be most needed out of all the future directions, as better automation and computational processing power in medical centers are integral to clinical implementation of artificial intelligence applications.

Potential future developments in artificial intelligence applications to clinical medicine specifically cannot be acknowledged without addressing the inherent limitations. For example, multiple studies in this field utilize small datasets, potentially using biased data, or are difficult to interpret in a clinical context (making decisions that have little to do with evidence-based medicine) [68]. Other potential limitations are the normal differences in anatomy among patients and correctly using previous imaging in AI processes [68]. Such limitations can lead to artificial intelligence models unfit for actual clinical medicine practice [68]. In addition, it is important to note that these AI tools cannot be a substitute for a qualified radiologist, but rather a tool to help clinicians practice due to their inability to provide complete clinical care [69].

In short, there is likely potential for clinical benefit within several applications of radiomics and AI to spinal tumors. Table 2 summarizes key findings on the current developments and future of the fields for specific applications to spine tumors.

## 7. Conclusions

Recent developments in the fields of radiomics and artificial intelligence show promise in improving the diagnosis and treatment of spine tumors. Collaborative, continued, and standardized efforts to validate and refine these tools across diverse populations and clinical settings are necessary to translate these advancements into clinical practice.

## Figures and Tables

**Table 1:** Summary of the search methodology and requirements for included studies.

Methodology Steps	Description
Literature Search	PubMed, EMBASE, and Scopus
Inclusion Criteria	Full-text articles published in English were included, with no specific timeframe. Original articles, clinical studies, case reports and series involving humans were included
Exclusion Criteria	Abstracts and unpublished studies were excluded
Search Terms	(“radiomics” OR “radiomic features” OR “imaging biomarkers”) OR (“machine learning” OR “artificial intelligence” OR “deep learning” OR “predictive modeling” OR “neural networks” OR “support vector machines” OR “random forests”) AND (“management” OR “treatment” OR “therapy” OR “intervention” OR “surgical management” OR “radiotherapy” OR “chemotherapy”) AND (“spinal cord tumors” OR “spinal cord neoplasms” OR “intramedullary tumors” OR “extramedullary tumors” OR “spinal tumors” OR “spinal neoplasms” OR “spinal cord glioma” OR “spinal cord astrocytoma”)
Additional Search	A manual search was performed to identify references from recently published series and reports
Sample Size Requirement	No strict sample size requirement

**Table 2:** Key findings about the current status and future possibilities of applications of AI and ML models in spine tumors.

Application	Current Status	Future Possibilities
Risk Assessment and Treatment Planning	Researchers have created radiomics/AI models for treatment planning and risk assessment [18,30,31,32,33,34]. Other studies offer automated treatment planning [35] and AI-optimized treatment plans [36].	Integrations of these methods into clinical workflow can allow for better selection of patients and efficient treatment planning [33].
Predictive Models for Outcomes and Prognosis	Multiple studies have utilized predictive modeling and radiomics to predict outcomes [21,30,38,39,40,41,42,43,44,45,46].	Incorporation of outcome prediction into clinical workflow may help in informing the physician–patient shared decision-making process as well as potentially minimize adverse outcomes [39].
Radiomics	Incorporation of radiomics may offer new insights into spinal disease [23,24].	Improvement of interpretability by creation of large, high-quality datasets available to the public and the adoption of universal standard workflows [24].
Role in Individual Tumor Types	Researchers have utilized radiomics and/or AI/ML to study specific details about individual tumor types [2,5,6,47,48,49,50,51,52,53,54,55,56,57,58,59,60,61,62,63].	Models specific to tumor type may allow for deeper insights into disease pathology and individualized outcomes for patients.

## Data Availability

No new data were created or analyzed in this study.
